# Effects of an Endocrine Disruptor Triclosan on *Ruditapes decussatus*: Multimarker and Histological Approaches

**DOI:** 10.3390/ani13030402

**Published:** 2023-01-25

**Authors:** Amira Added, Noureddine Khalloufi, Abdelhafidh Khazri, Abdel Halim Harrath, Lamjed Mansour, Saber Nahdi, Fehmi Boufahja, Waleed Aldahmash, Abdulwahed Fahad Alrefaei, Mohamed Dellali

**Affiliations:** 1 LR01ES14 Laboratory of Environment Biomonitoring, Coastal Ecology and Ecotoxicology Unit, Faculty of Sciences of Bizerte, University of Carthage, Zarzouna 7021, Tunisia; 2 Zoology Department, College of Science, King Saud University, P.O. Box 2455, Riyadh 11451, Saudi Arabia; 3 Biology Department, College of Science, Imam Mohammad Ibn Saud Islamic University (IMSIU), Riyadh 11623, Saudi Arabia

**Keywords:** *Ruditapes decussatus*, endocrine disruptors, triclosan, biomarkers, histopathology

## Abstract

**Simple Summary:**

The multiple effects of endocrine disruptors on aquatic invertebrates have rarely been assessed based on integrative approaches, despite the clear advantage of exploring histology in association with traditional acute toxicology tools. Applying such approaches is critical in significantly reducing health risks for human populations that regularly consume seafood such as bivalves. The data presented here support the use of the European clam *Ruditapes decussatus* as a biological indicator of the presence of triclosan in seawater, and demonstrate the usefulness of biomarker assessment and histological features in determining environmental thresholds and government guidelines.

**Abstract:**

The aim of this work was to study the ecotoxicological effects of an endocrine disruptor triclosan on the clam *Ruditapes decussatus*. The bivalves were exposed to three concentrations of this biocide (C1 = 100 ng/L, C2 = 200 ng/L and C3 = 500 ng/L) for three and seven days. The impact was assessed at the gills and digestive glands, through activities of an antioxidant defense biomarker (Gluthatione *S*-Transferase, GST), a damage biomarker (Malondialdehyde, MDA), and a neurotoxicity biomarker (Acetylcholinesterase, AChE). Furthermore, histological traits were approached in different organs to evaluate any possible alteration induced by triclosan. It appears from this study that both gills and digestive glands responded discernibly to triclosan and effects were concentration-dependent. The stressed clams showed a significant increase in their GST and MDA activities in gills and digestive glands compared to controls for both time slots considered. In turn, the AChE activity was clearly inhibited in both organs in a time dependent way. The histological study made it possible to observe several structural pathologies caused by triclosan in the gills and the digestive gland. These alterations consisted mainly of inflammatory reactions, malformations of the lamellae and fusion of the gill filaments, degeneration of the connective tissue, and the erosion of the gill cilia with the appearance of certain severe alterations (cell necrosis and apoptosis), which can thus cause a malfunction of the gills and eventually lead to a reduction in oxygen consumption and a disruption of the osmoregulation for bivalves. Alterations in the digestive gland have also been detected, mainly by epithelial alterations, thinning of the tubules, and alteration of the basal cell membrane which can impair the ability of clams to absorb food. At germinal cells, several damages were observed in the oocytes which probably disturbed the reproductive function and the fertility of the clams. The damages observed in female gonads were caused by the cytolysis of a large number of oocytes through autophagy and necrosis at 200 ng triclosan/L. Moreover, at 500 ng triclosan/L, hemocytic infiltration was observed in acini and apoptotic bodies reflected in the fragmentation of more than 90% of oocytes.

## 1. Introduction

In recent years, degradation of the aquatic domain increased with the increase of pollution magnitude, the appearance of invasive taxa, and diversity loss [1]. This seems to be generated in one way or another by the development of human societies through agricultural, urban and industrial waste [2]. A decrease in the fishery production of these areas has been recorded throughout the world, and particularly in coastal lagoons [3] such as the Bizerte lagoon, a vulnerable and relatively isolated ecosystem in the extreme north of Tunisia.

In the aquatic environment, numerous chemicals (metals, polycyclic aromatic hydrocarbons (HAPS), polychlorobiphenyls (PCBS), biocides and organochlorine pesticides (OCPS)), mostly of anthropogenic origin, constitute a risk for human health after consumption of contaminated seafood such as molluscs or shrimps, fish, crabs, etc. [4]. During the last two decades, certain contaminants emerged and worried the scientific community because of their unknown or less documented impact and/or their new detection in aquatic areas becoming possible by the continuous development of new analytical techniques. 

Among the emerging contaminants of interest, endocrine disruptors became a source of environmental concern [5]. The rivers and lentic ecosystems (lagoons, lakes, etc.) are contaminated by endocrine disruptors through their use in agriculture (pesticides, hormones from intensive farms, etc.), and industrial (effluents of pulp plants, etc.) or untreated domestic wastewater [6]. Furthermore, in some cases, the wastewater plant outcomes are still contaminated by environmental xenoestrogens (such as pesticides, polychlorinated biphenyls, and plasticizers) and environmental anti-androgens (such as linuron and vinclozolin) which could not be eliminated during the treatments [7]. Among endocrine disruptors, triclosan is present in many current consumed products due to its common use as an antibacterial agent, and is already suspected of multiple harmful effects on human health and the environment. Triclosan has been measured in benthic sediments from aquatic habitats at concentrations high enough to be further bioaccumulated in plants and animals. This biocide was found in concentrations ranging from 48.3 to 226 ng/g dry weight in surface sediments near the outfalls of municipal wastewater treatment plants in Nanjing province, China [8].

Several studies have been conducted on the effects of xenobiotics on aquatic organisms, and particularly bivalves, mostly because of their sessile filter feeder life style [9]. Thus, bivalves can bioaccumulate large amounts of organic pollutants in their tissues, leading to symptoms of cumulative pollution [10]. Moreover, their global distribution and low acquisition costs make bivalves common materials in ecotoxicology experiments to assess multiple adverse effects of bioaccumulation at ecophysiological measured endpoints [11]. However, (1) the chronic toxicity of triclosan, despite its high ability to accumulate on suspended and sediment particles and to spread and concentrate in aquatic feeding networks, was understudied [12], and (2) only the physiological features are generally explored. The triclosan alters the metabolic capacity of bivalves, glycogen and protein contents, as well as the antioxidant and biotransformation defence mechanisms [13]. References [14] and [13] performed laboratory experiments to study the physiology and metabolic rate changes in *Ruditapes decussatus* and *R. philippinarum* following their exposure to different temperatures and pH conditions in association to triclosan. The results showed that both species of bivalves reduced their growth and reproductive potential in the presence of triclosan. The current work aims to fill the knowledge gap regarding the effects of triclosan on aquatic invertebrates by focusing on its chronic toxicity to a species of bivalve, which dwells in water and sediment milieus (i.e., *R. decussatus).* The current study tries to answer the following questions: (i) Is triclosan harmful to clams? (ii) If yes, what is the threshold, based on biochemical and histological data? We expect that this biocide will have a significant impact on biochemical biomarkers in *R. decussatus*, with no idea if the effects of trisclosan would be seen on histological levels, since these are totally recently explored axes.

## 2. Material and Methods

**Figure 1:**
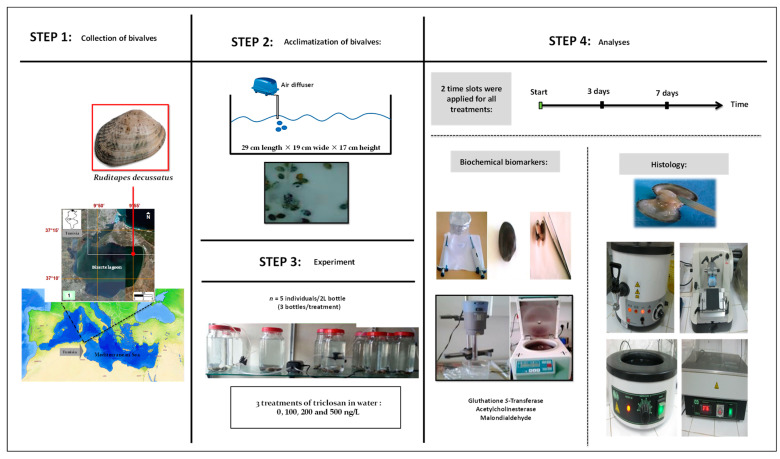
Graphic summary of steps and working methodology.

A graphical summary of the timeline and experimental design, as well as performed analyses, is given in Figure 1. Details of all steps and methods followed are presented below.

### 2.1. Collection Site of R. decussatus

The clams were collected manually at the site Menzel Jemil (37°13′11.14″ N, 9°56′6.80″ E) in Bizerte lagoon, Tunisia. They were put in a cooler containing water from the sampling site and immediately transported to the laboratory. Upon arrival, the collected individuals were placed in tanks (29 cm length × 19 cm wide × 17 cm height) for a week of acclimatization at 18 °C with a light/dark cycle of 12h/12h [15]. To ensure the survival of the clams, ventilation using air pumps was applied and the water was replaced every two days [15]. No artificial food was added [15]. 

### 2.2. Experimental Set-Up

Triclosan was purchased from Sigma-Aldrich, St. Louis, Mo, USA (CAS No.: 3380-34-5). It was first dissolved in ethanol to test the effects on clams of 4 treatments including 3 targeted concentrations of triclosan, 100 ng/L (C1), 200 ng/L (C2), and 500 ng/L (C3), and an untreated control with only sea water (Ut). All treatments were applied in triplicate, with 5 individuals per 2 L glass bottles or ‘microcosms’. All manipulations were carried out under well controlled conditions of 18 °C and a photoperiod of 12h/12h [16]. These authors mentioned that triclosan is highly available for aquatic organisms because of its low photodegradability, non-volatility (5.3 × 10^−4^ Pa at 20 °C) and relatively high solubility in water (10 mg/L at 20 °C). Triclosan was found in marine, coastal and estuarine waters. According to [14] and references within, this biocide may reach concentrations as high as 2000 ng/L in the wastewaters of many countries in Europe, North America, Australia and China. In contrast, concentrations as low as 10–124.1 ng/L were found in pristine marine waters in Portugal [14]. The concentration C1 (i.e., 100 ng/L) was chosen to be representative for the low concentrations range of triclosan, whereas the higher concentrations, namely C2 (i.e., 200 ng/L) and C3 (i.e., 500 ng/L), were obtained by multiplying C1 by 2 and 5, respectively.

No special diet protocol was followed during the experimental period and water was renewed in the aquaria early in the morning every two days (i.e., 1st, 3rd, 5th, and 7th) in all glass bottles (i.e., controls and treatments). 

### 2.3. Biochemical Biomarkers Study

At the end of the current study, the enzymatic activities of three biochemical biomarkers were analysed at the gills and digestive glands of the clam *R. decussatus*: glutathione *S*-transferase (GST), acetylcholinesterase (AChE), and malondialdehyde (MDA). In addition, histological alterations were studied at both organs considered.

First of all, the clams were detached from their shells; the gills and the digestive gland of each animal were, thus, separately placed in marked cold pillboxes. The dissection was made in the ice (4 °C) to avoid distorting the proteins. The extraction of the latter was carried out in a buffer composed of 10 mM Tris/HCl, pH 7.4, containing 500 mM sucrose, 1 mM EDTA, and 1 mM PMSF. The tissue homogenization was processed in a T25 Ultra-Turrax. The homogenate obtained was then centrifuged at 9000 g for 30 minutes at 4 °C. The cytosolic extract (S9) was finally divided in several Eppendorf tubes then kept at a temperature of −80 °C for subsequent biochemical analysis.

The amount of total proteins was estimated using the Bradford assay [17]. The product was quantified based on the colorimetric change of Coomassie Brilliant Blue G-250 stain after complexation with basic amino acids and the hydrophobic residues of the amino acids present in the proteins. The measurement of changes in optical density was realized using a Beckman DU500 spectrophotometer at 595 nm by considering bovine serum albumin (BSA) as a standard protein.

The glutathions *S*-transferases (GST) are a family of mainly cytosolic isoenzymes. The measurement of their specific activity was carried out as reported in [18]. The protocol described by these authors consists of reacting, in a phosphate medium (100 mM, pH 8), the cytosolic extract (S9), with the chloro-2,4-dinitrobenzene (CDNB) as the substrate, in the presence of a Glutathione (GSH) as a cofactor. The reaction leads to the formation of a complex CNDB-GSH, with maximum absorption of 340 nm. The variation in optical density due to the appearance of the CDNB-GSH complex is measured using a UV/visible Beckman DU500 spectrophotometer. One unit of GST was defined as the quantity of glutathione conjugate produced by 1 mM GSH and CDNB/min per mg protein.

The acetylcholinesterase (AChE) activity was determined by adopting the protocol of Ellman et al. (1961). This method is based on the hydrolysis of a choline ester (acetylthiocholine) by acetylcholinesterase to release thiocholine. The produced thiocholine reacts with 5-5 dithio-bis (2- nitrobenzoate) (DTNB) to form 5-thio-2 nitrobenzoate (TNB), a yellow product with absorption at 412 nm. The colouring intensity is proportional to the AChE amount. After incubation at 25 °C, the absorbances were measured in samples by using a microplate reader at a wavelength of 412 nm [19].

*Malondialdehyde* (MDA) is determined according to the method of [20] as a commonly used biomarker for lipid peroxidation. The protocol followed is based on an acidic liberation of the MDA. Thus, the aldehydes react with the thiobarbituric acid (TBA) at 95 °C in aerobic conditions (pH 3.4) to form a chromogenic condensation pink product, and optical density was read spectrophotometrically at 532 nm. It must be mentioned here that the protocol presented above measured contents of MDA and also other minor forms of aldehydes.

### 2.4. Histopathological Study

The valves of clams from control and treatment glass bottles were opened by incision of the adductive muscle with a scalpel. The gills and digestive glands were carefully removed and stored in pillboxes until further histological analysis. The preparation of samples for the microscope analysis was carried out according to [21]. In order to preserve the cell structures, the fixation was carried out in 10% Neutral Buffered Formalin (NBF), whereas the dehydration was carried out using a series of stepwise-increasing concentrations of ethanol and toluene. The paraffin treated samples were then cut in 5 µm thick sections using a manual microtome and set in slides in albuminous water. After staining using hematoxylin and eosin (H&E), the samples were examined under an optical microscope with a CCDET incorporated camera. 

### 2.5. Data Processing

The one-way analysis of variance (ANOVA) was necessary for global comparison between treatments. If significant differences were registered, ANOVA was followed by Tukey’s HSD post hoc test for pair-wise comparisons, particularly between traits of control clams and those exposed to the three levels of triclosan. Both tests mentioned above are parametric and require two preliminary assumptions: normality and homogeneity of variance. Thus, before applying them, our data were tested using tests of Kolmogorov–Smirnov and Bartlett, respectively [22]. All analyses were performed using Statistica v8.0 software and the threshold of significance was set at *p* < 0.05.

Histopathological alterations have been evaluated semi-quantitatively by the classification of the severity of the observed lesions [(level 0 (−), 0.5 (+/−), 1 (+), 2 (++), and 3 (+++)]. A damage index has been calculated to eventually allow the comparison of histopathological responses of the clams to the different concentrations of triclosan [100 ng/L (C1), 200 ng/L (C2), and 500 ng/L (C3)]. The damage index is an average arithmetic value obtained from the semi-quantitative evaluation of the lesions [23].

## 3. Results

### 3.1. Biochemical Biomarkers

The effects induced by triclosan on the clam *R. decussatus* were assessed by measuring the activities of three biochemical biomarkers: the glutathione *S*-transferase (hereafter GST), a biomarker of defence against the oxidative stress, the malondialdehyde, a biomarker of the damage degree in lipids (hereafter MDA) and acetylcholinesterase, a biomarker of neurotoxicity (hereafter AChE).

#### 3.1.1. Glutathione S-Transferase (GST) Activity

**Figure 2:**
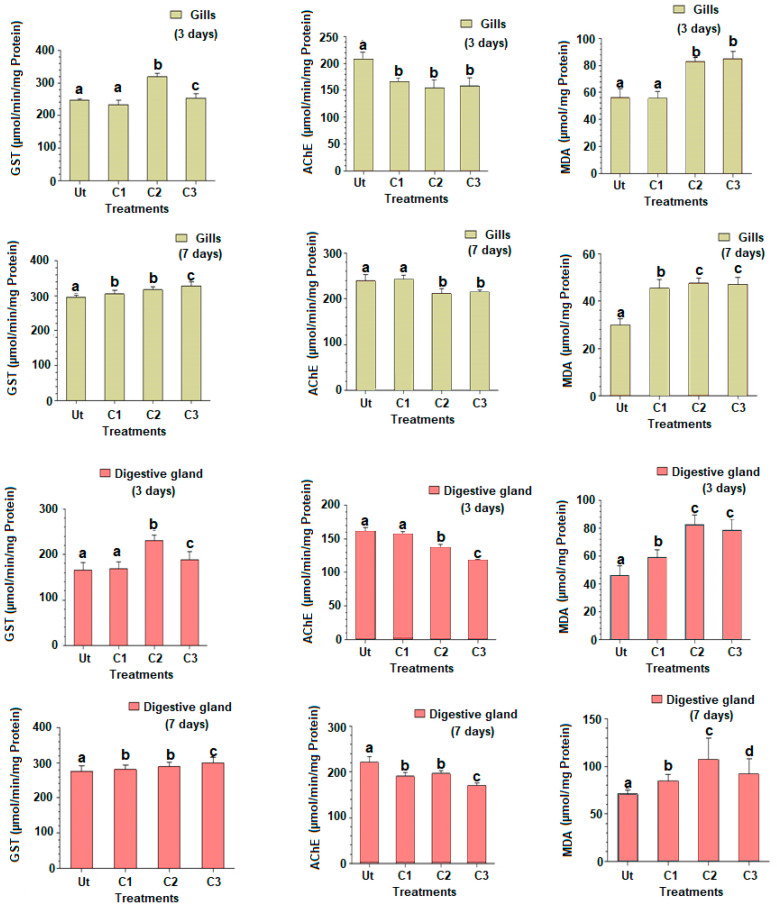
Gluthatione *S*-transferase (GST) and acetylcholinesterase (AChE) activities and malondialdehyde rate (MDA) in gills and digestive glands of the European clam *Ruditapes decussatus* after exposure to triclosan (C1 = 100 ng/L et C2 = 200 ng/L et C3 = 500 ng/L) during three and seven days in comparison to the untreated controls (Ut). Different letters (a, b, and c) above bars indicate significantly differences from controls represented by ‘a’ after multiple comparisons using Tukey’s HSD test.

The GST activities for the two time slots (three and seven days) are provided in Figure 2, and expressed as mean ± SD. Following three days of exposure to triclosan, the changes in GST activities showed similar changes in the gills and digestive glands. The GST activity was similar in C1 compared to the control (Tukey’s HSD test: *p*-value = 0.921), but increased significantly in C2 and C3 for both organs (Tukey’s HSD test: *p*-values < 0.001), to a lesser degree in C3 (Tukey’s HSD test: *p*-values < 0.05) (Figure 2). The exposure of clams to triclosan after seven days revealed that both organs responded, once again similarly, with an increased GST activity in a dose-dependent way for all concentrations tested and the two organs studied (Figure 2).

#### 3.1.2. Acetylcholinesterase Activity (AChE)

The AChE activity in gills after three days showed a significant decline in C1 compared to control (Tukey’s HSD test: *p*-values < 0.001), but a similarity with treatments C2 and C3 (Figure 2). In the digestive gland, the AChE activity significantly decreased in C2 and C3 compared to C1 and control. After seven days of exposure to triclosan, the AChE activity in gills was significantly reduced in C2 and C3 compared to C1 and control treatments (Tukey’s HSD test: *p*-values < 0.001) (Figure 2). A similar response was observed for the digestive gland, with a significant reduction in the AChE activity in C1, decreasing with the triclosan dosages used (Tukey’s HSD test: *p*-values < 0.001).

#### 3.1.3. Malondialdehyde Rate (MDA)

The MDA content in gills increased in treatments C2 and C3 compared to C1 and control after three days (Figure 2). The increase of the MDA rate was also registered in the digestive gland, but starting with C1, in a time-dependent effect (Tukey’s HSD test: *p*-values < 0.001). After seven days of exposure, an increase in the MDA rate was found in both organs, starting with C1 (Tukey’s HSD test: *p*-values < 0.01) (Figure 2). A statistically marginal reduction of the MDA rate was observed in C3 compared to C2 for both organs (Tukey’s HSD test: *p*-value = 0.041) (Figure 1). 

### 3.2. Histological Study

#### 3.2.1. Observed Pathologies

**Figure 3:**
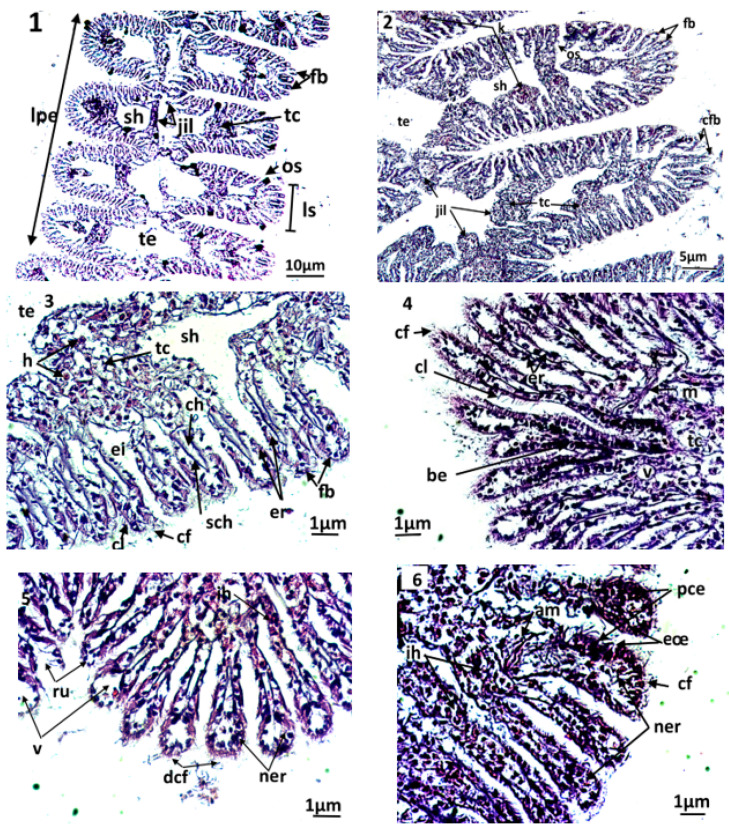
Microphotography of gill strips ((**1**,**2**) general appearance; (**3**): control clams) and gill filaments of an exposed clam to triclosan (C3) (**4**–**6**). nt; necrotic tissue; ac: alteration of eyelashes; am: alteration of gill muscle fibres; be: basophilic epithelium; ch: hemolymphatic canal; cf: frontal eyelashes; cl: lateral eyelashes; eœ: edema of the epithelial tissue; fb: gill filaments; jil: interlamellary junction; ih: hemocytic infiltration; lpe: external primary gell lamella; ls: secondary gell strips; os: ostiole; pce: pyknotic epithelial cells; ne: necrotic epithelium; ner: necrosis of respiratory epithelium; nt: necrotic tissue; ru: rupture; sch: chitinous skeleton; sh: hemolymphatic sinus; tc: connective tissue; te: water tubule; v: vacuolization.

The gills of the clam (Figure 3(1–6)) comprise two primary strips, the external and the internal gill strips. Each primary strip comprises, in turn, a series of folds or the secondary strips, which are made of gill filaments. A gill filament consists of a layer of epithelial cells (i.e., the respiratory epithelium) and is supported along the filament by two internal stems (i.e., the chitinous skeleton).

The filaments carry the gill eyelashes (i.e., frontal, lateral, and frontal/lateral). The inter-lamellar space is occupied by the water tubules, separated by inter-lamellar junctions, but which can communicate with the pallial cavity by ostioles. A connective tissue exists at the basis of the gill filaments, creating the hemolymphatic sinuses. Both gill strips are linked by connective tissue.

By the end of the experiment, the histological features of control clams and clams exposed to triclosan were studied. In untreated clams, the gills were normal, with well-defined and uniformly arranged gill strips (Figure 3(2–3)). The filaments of each gill lamella were of similar length and thickness, covered with unrealized gill eyelashes. The density and the appearance of hemocytes in the connective tissue and hemolympatic sinuses were normal.

The gills of clams exposed to Triclosan showed differences in their architecture and various damages in the respiratory tissues (Figure 3(4–6)). Although they kept a normal architecture, the gills of individuals exposed to C1 (100 ng/L) exhibited a slight structural change compared to the controls, evidenced by greater hemocyte infiltration rate, an elongation of filaments, a rare vacuolization of the connective tissue, a smaller inter-filamentous space, and a slight dilation of the hemolymphatic canal (Figure 3(4)).

In gills, the most important histological modifications were observed following the exposure of clams to the highest concentrations of trisclosan, namely the C2 and C3 treatments (Figure 3(5–6)). In these clams, strong hemocytic infiltration was observed. In C2 treatments, the gills showed severe dilation of the hemolymphatic canal at the end of the filaments, compared to C1. Such symptoms precede the degeneration and the necrosis of the respiratory epithelium. Moreover, the slimming of the epithelium led to ruptures of the end of the filaments. For clams exposed to the highest concentrations of triclosan, C3, the damages observed in the respiratory tissues were intense. Thus, significant inflammations such as hemocytic infiltration, edema, mainly at the end of filaments, and necrotic and apoptotic epithelial cells were observed. Additionally, alteration of the muscle fibres were found in gills, paralleled to ruptures in the chitinous skeleton. The gill filaments were hypertrophied, with dilation of the hemolymphatic channels and desquamation of the filamentous eyelashes (Figure 3(6)).

**Figure 4:**
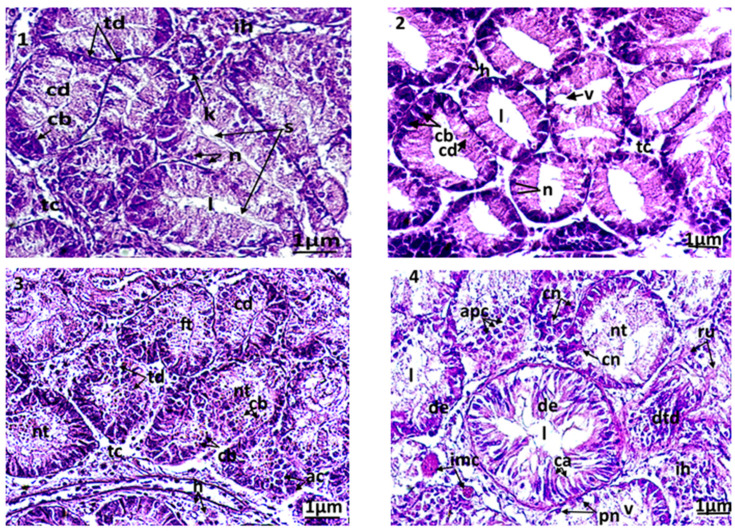
Microphotography of digestive tubules. (**1**): control clams; (**2**): clam exposed to triclosan (C1); (**3**): clam exposed to triclosan (C2); (**4**): clam exposed to triclosan (C3). ac: apoptotic cells; cb: basophilic secretory cells; cd: digestive cells; cn: necrotic cell; de: epithelium degenerated; dtd: digestive tract degenerated; ft: fusion of tubules; h: hemocytes; l: light of the digestive tract; n: normal nucleus; nt: necrotic tissue; pn: pyknotic nucleus; ru: rupture of digestive tubules; s: secretion; v: vacuolization.

Several histopathologic damages were registered in the digestive glands of the treated clams compared to control. The control clams had healthy digestive tubes, with low hemocytic infiltration levels in the connective tissue (Figure 4(1)). The digestive tubes showed a pseudostratified epithelium, comprising abundant eosinophilic digestive cells and basophilic secretory cells, stained in blue by haematoxylin. The light of the digestive tubes in control bivalves was closed or slightly opened. In animals exposed to triclosan, several microscopic changes were observed compared to control (Figure 4(2–6)). The first anomaly observed was the dilation of the light in the digestive tubes, which was proportional to the concentration of the tested biocide. In C1, the dilation of the digestive light and vacuolization occurred slightly, but with no notable alterations of the tubes. The latter kept their normal epithelium in the absence of an abnormal infiltration of hemocytes (Figure 4(2)). More significant damages were observed at high concentrations of triclosan (C2 and C3): a fusion of the digestive tubes, a degradation of the epithelium, often paralleled by necrosis, but also apoptosis of the digestive cells (Figure 4(3–4)). In the connective tissue, a high infiltration of hemocytes was observed. The most severe disturbances were seen at high, strong microscopic magnification in secretory cells, where a condensation of the chromatin and a fragmentation of the nucleus occurred.

**Figure 5:**
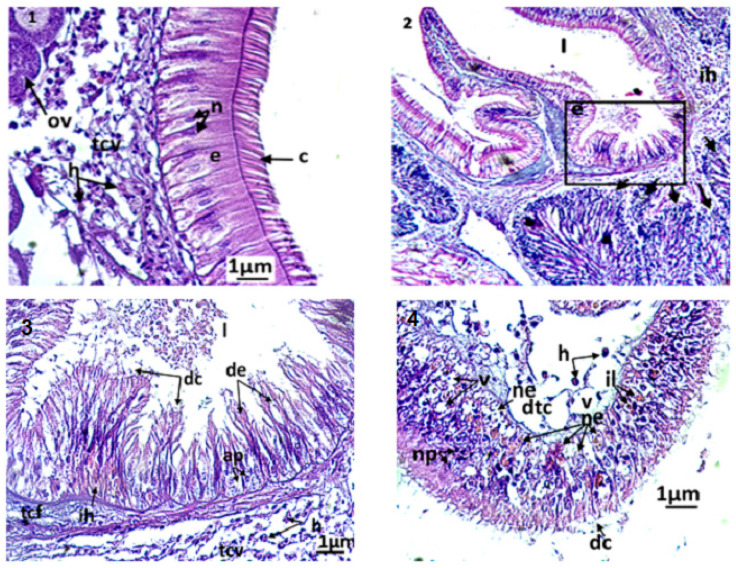
Micrographs at the digestive tract of *Ruditapes decussatus* exposed or not to triclosan. (**1**): Microphotography of the intestinal epithelium of control clams. C: eyelashes; e: pseudostratified epithelium; h: hemocytes; ov: oocyte; n: normal nucleus; tcv: vesiculated connective tissue. (**2**,**3**): microphotography showing the stomach and male acini of a treated individual (C3, 3 days). The arrows indicate the degeneration of the acini (desquamation of the basal membrane). ap: apoptotic nuclei; ih: hemocytic infiltration; l: stomach light; tcf: fibrous connective tissue; de: degenerated epithelium; dc: cellular debris. (**4**): dtc: degeneration of the connective tissue; h: hemocyte; il: inclusion of lipid granules; ne: necrosis of the epithelium; np: pyknotic nucleus; v: vacuolization.

In the stomach and the intestine, the same type of damage was observed as was found in the digestive tissues. In detail, the intestine and the stomach of the control individuals has a ciliated pseudostratified epithelium. A normal tissue at the connective tissue (Figure 5(1)), non-eroded eyelashes, and an epithelium without anomalies, with normal basophilic and digestive nuclei cells with homogeneous chromatin, were also observed. In turn, severe damages were found, in particular following the exposure of clams to C3, with important hemocytic infiltration in the connective tissue, an infiltration of phagocytic cells at the intestinal epithelium by diapedesis, a hypertrophy of the cell nucleus of the epithelium, necrosis and apoptosis inducing a vacuolization, degeneration and a loss of the normal architecture, and an erosion of the eyelashes of digestive cells (Figure 5(2–4)).

**Figure 6:**
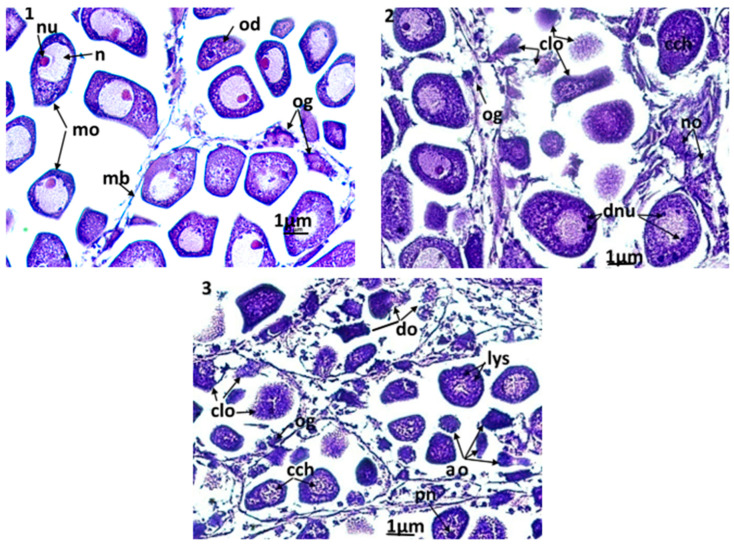
Microphotographs of the female gonad (×400). (**1**): control clams, the acini are filled with normal mature oocytes; (**2**): clams exposed to triclosan (C2), (**3**): clams exposed to triclosan (C3). ao: apoptotic oocytes; cch: condensation of chromatin; clo: cytolysis of oocytes; do: degeneration of oocytes; dnu: nucleolic division; lys: lysosomes; mo: mature oocyte; n: nucleus; no: necrotic oocyte; nu: nucleolus; od: oocyte in development; og: ooogonia; pn: pyknotic nucleus.

The clams used in the current histological study were adults and showed an advanced stage in gametogenesis. In both sexes, mature gametes (i.e., spermatozoa and oocytes) were observed. In males, it was difficult to see a clear damage caused by the exposure to triclosan. In turn, the disturbances were more visible in the female gonads (Figure 6(1–3). Among the control clams (Figure 6(1)), the female gonads had acini containing oogonia, oocytes under development, and mature oocytes with cytoplasm rich in eosinophilic vitellin. In these cells, the nucleus occupied a large part of the cytoplasm and contained an excentric nucleolus with eosinophilic affinity. The chromatin in the nucleus of the mature oocytes was homogeneous and unconnected. In specimens exposed to the lowest triclosan concentration (C1), there was no notable damage. However, for higher concentrations of triclosan (C2 and C3), significant changes in gametes were observed; a slight condensation of chromatin and a division of nucleoles (Figure 6(2)) was observed in clams used in C2 treatments (i.e., 200 ng/L). Moreover, a discernible number of oocytes that underwent cytolysis through autophagy and necrosis, that started with the appearance of lysosomes in the cytoplasm, were also present. Under this level of stress, some almost ‘healthy’ oocytes were still present in each acinus, but the acini had altered membranes. For even stronger concentrations (C3 = 500 ng/L), the damage was more intense (Figure 6(3)) since more than 90% of oocytes degenerated for certain acini. There was significant hemocytic infiltration even in acini and apoptotic bodies resulting from the fragmentation of oocytes.

#### 3.2.2. Index of Damage (ID)

**Figure 7:**
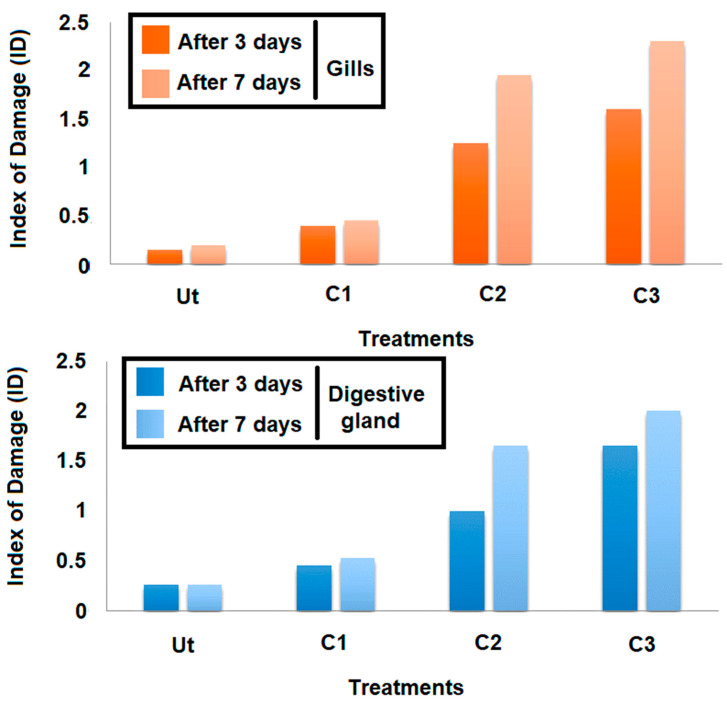
Variation of the Index of Damage (ID) at the gills as a function of concentration and exposure time.

The cellular damage induced by the triclosan was assessed by using the Index of Damage (ID). Based on the results obtained (Figure 7), it occurred that this index evolved in a linear manner, depending on the concentration tested for both time slots adopted (three days and seven days) and organs considered (Figure 7). Moreover, the comparison of IDs for the same concentrations after three and seven days showed that this index increased according to the exposure duration whatever the concentration applied. The intensities of each lesion noticed in gills and digestive glands, and the damage index (ID), are given in Table 1 and Table 2, respectively. After three days of contamination, a hemocyte infiltration was always observed in clams exposed to C3, but less frequently in C2. Under the highest concentration of triclosan (C3), many pathological alterations were also more frequent compared to the control treatment. However, in other concentrations (C1 and C2), cellular and tubular necrosis, an alteration of connective tissue, an elongation of tubes, the secretions, and the vacuolization were recorded. 

## 4. Discussion

The assessment of biological responses in sentinel taxa exposed to environmental stressors is considered a preventive approach to protect coastal ecosystems and further human health [24,25,26]. Numerous authors have mentioned the importance of using different biomarkers simultaneously in biomonitoring and ecotoxicological studies when using bivalves [27,28]. However, none have tried to make a more global approach by also exploring histopathological alterations. 

### 4.1. Does Triclosan Affect the Biomarker Activities in R. decussatus?

In the current study, the analysis of biomarkers’ activities was carried out in two organs in the European clam *R. decussatus*, the gills and digestive glands, due to their principal roles in the absorption and accumulation of contaminants [29]. 

After exposure to triclosan for three and seven days, the treated clams showed an increase in the GST activity measured in the gills and the digestive gland compared to the controls. A notable increase in GST activity was previously reported in *R. decussatus* by [30], and in other bivalves’ taxa such as *Mytilus edulis* and *M. galloprovincialis* following their exposure to polycyclic aromatic hydrocarbons (PAHs) [16,31]. Similar patterns of the GST activity were also found by [32] in gills when mussels from the coasts of Galicia (Spain) were reared in contaminated water by PAHs and polychlorinated biphenyls (PCBs). GST *S*-Transferase glutathiones are enzymes of detoxification (phase II) capable of combining a reduced glutathione molecule (GSH) with a given xenobiotic in order to make it more soluble, and therefore more easily excretable by the organisms [33]. It is also considered as a defence biomarker against oxidative damage and lipid peroxidation products [34]. The exposure of clams to triclosan concentrations shows an increase in GST activity, indicating an induction of the system. Such an increase in the activity of this enzyme implied an activation of the defence system for metabolization in clams following their exposure to the biocide in question.

The AChE activity has been widely used as a biomarker of neurotoxicity in bivalves [35], especially in the case of biocides, organophosphorus pesticides and carbamates [6,36]. In our case, the activity of this enzyme was significantly reduced in clams reared in experimental units contaminated by triclosan. This result is in accordance with those of [37], who found an inhibition of the AChE activity in *R. decussatus* collected from the lagoon of Ria Formosa (Portugal) where diverse contaminants (metals, PAHs, tributyltin) are present. Similarly, a similar trend in AChE activity was observed in the gills of the clams *R. philippinarum* collected from polluted sites in Venice lagoon (Italy) [38] and *Scrobicularia plana* picked from the most polluted sites in a swamp in Spain [39]. Moreover, the inhibition of the AChE acitivity under stress characterized animal groups other than molluscs, for example in the case of the polychaete *Nereis diversicolor* [39] and the crab *Carcinus maenas* [40]. The explanation of [41] could be adopted for the results above. These authors suggested that contaminants may influence the AChE activity in sentinel organisms by altering the synthesis path of the enzyme itself, or simply by affecting the general health of the organism and thus reducing the production rate of the enzyme. 

Environmental contamination leads to the generation of Reactive Oxygen Species (ROS), which can modify biological parameters and generate oxidative stress in organisms [42]. The oxidative stress occurs when the production of ROS exceeds the capacities of the antioxidant defence system. Lipid peroxidation has been herein estimated in *R. decussatus* through the MDA rate. A significant and progressive increase in the MDA accumulation was found during the two exposure periods chosen (i.e., three and seven days), most probably because of the presence of triclosan. Indeed, the trend obtained supported a close relationship between the MDA level that shows the magnitude of lipid peroxidation and the concentration of tricolsan. Such a finding is in accordance with the literature, since xenobiotics are known for their potential to cause lipid peroxidation in cell membranes [26,43,44]. For example, the clam *R. decussatus* transplanted into the lagoon of Ria Formosa (Portugal) showed increased levels of lipid peroxidation due to the high contamination by PAHs there [45]. High levels of lipid peroxidation have been observed, too, in the crab *C. maenas* collected from the Bizerte lagoon, and are linked to metallic contamination [40]. 

The deleterious effect of triclosan on the metabolism of oxygen, by acting on the respiratory channel, was observed on the African clawed frog *Xenopus laevis* [46,47]. Other studies [48,49] showed that the exposure to triclosan induces in several aquatic species neurotoxic and endocrine disturbances, and affects the functioning of numerous receptors and intracellular Ca^2+^ channels, causing the development of oxidative stress and cell apoptosis. These studies also showed that the triclosan had a direct effect on the lipid membranes, by modifying their permeabilization, a reaction observed also in artificial lecithin liposomes. This biocide also inhibits the activity of mitochondrial respiratory chain complexes (both in vitro and in vivo), leading to the overproduction of ROS (primarily superoxide) and lipid peroxidation and accumulation of MDA. The latter compound was found both in vitro, in isolated mitochondria, and in vivo, particularly in amphibians [47]. 

### 4.2. Are Histological Features Modified by Triclosan?

For bivalves, the gills and the digestive gland play an important role during the uptake, absorption and digestion of food. Consequently, the exposure of bivalves to triclosan present is expected to alter their feeding capacity, and thus compromise their growth and reproduction [50]. In the current work, the histopathology of clam gills exposed to the different concentrations of the triclosan (C1, C2, and C3) revealed several structural alterations. Indeed, clams exposed to the biocide for seven days have gills with stronger alterations compared to those under stress only for three days. The histologic investigations indicated alterations of the gill strips, which could have induced a dysfunction of gills, subsequently leading to the reduction in oxygen consumption and disruption of the osmoregulatory function in bivalves [51,52]. To be precise, the histopathological alterations registered consisted mainly of inflammatory symptoms (hemocytic infiltration, edema, and hypertrophy), malformations of the strips in gill filaments, degeneration of the connective tissue, and the erosion of the gill eyelashes with the appearance of certain severe alterations in the highest concentration of triclosan (cell necrosis and apoptosis). These changes in the respiratory tissue will lead to a progressive loss of the biological functions of the gills (i.e., gas exchanges and ionic regulations). Similar changes have been observed in the mussel *Perna viridis*, collected in the Ennore estuary located on the south–eastern coasts of India, with hemocytic infiltrations, a fusion of the gill filaments, and epithelial alterations [52]. Ciliary erosion is among the most frequent alterations in the gills [53]. It corresponds to a loss of lamellar eyelashes, which are involved in the filtration of suspended particles present in inhaled water, followed by the selection and transportation to the digestive tubes [53]. Thus, it appears that the alteration of eyelashes and gill filaments may affect both the respiration and the feeding processes of the clams, and subsequently impact their growth and productivity [51,52,53,54,55,56].

The semi-quantitative assessment of histopathological lesions has shown that the severity of damage in gills was proportional to the concentration of the triclosan used and the duration of exposure. The occurrence of histopathological alterations has been reported in published works in the gills of the clam *R. philippinarum* and the crab *C. maenas* collected from contaminated sites by metals [57,58]. The histopathological alterations observed in our study are similar to the alterations found in *R. decussatus* collected in a contaminated site on the southern coasts of Portugal [59], and in *R. philippinarum* experimentally exposed to metals (Cd, Cu, and Zn); [60,61] also reported necrosis and inflammation are the most common pathologies found in oysters collected at the coastal areas of Brittany (France) after the Cadiz Amoco oil spill. 

In bivalves, the digestive gland plays an important role in digestion and constitutes the main organ of accumulation and detoxification for several types of contaminants (PAHs, PCBs, metals, etc.) [52]. The clams exposed to triclosan for three and seven days presented severe alterations in the structure of digestive tubules and epithelial cells. At the tubules, pathologies varied from slight alterations (dilation of tubular light, vacuolizations, and hemocytic infiltration in the connective tissue) to severe alterations (degeneration of the digestive cells’ epithelium by necrosis and apoptosis). These changes are considered by several authors as protective mechanisms in order to maintain osmoregulation balance [62]. However, these alterations may lead, finally, to the degeneration of most digestive tubes and thus deeply affect the metabolism of the animal.

All the results obtained in the digestive gland suggest that histopathological alterations are closely linked to the accumulation of triclosan in the tissues of the clams. Several publications have shown similar findings in molluscs, or even other aquatic taxa. For example, results reported by [59] showed the numerous pathologies in digestive glands (formation of dilated light, the presence of cellular debris, hemocytic infiltration, the shrinkage of epithelial cells, the formation of tubular necrosis) of *R. decussatus* collected from sites under high anthropogenic pressure (commercial port and aquaculture farms) located in the southern coastal areas of Portugal. Reference [52] also mentioned severe histological alterations (hemocytic infiltration, epithelial alterations, slimming of tubules, alteration of the basal membrane, and atrophy) of the digestive gland in *P. viridis* collected in the Ennore estuary (India). According to them, these alterations are due to the accumulation of metals at the soft tissue of the mussels. Similar results have been found for the clam *Macoma calcarea* experimentally exposed to spilled oil [63] and the crab *C. maenas* collected from contaminated locations by trace metals in Bizerte lagoon, Tunisia [58].

The study of the histopathological response in the clam *R. decussatus* exposed to triclosan was followed by structural alterations in the gills and the digestive gland. These effects became worse with additional disturbances in germ cells after exposure to this endocrine disruptor. Indeed, in the female gonad, several damages have been observed to those affected oocytes: cytolysis, absorption, autophagy, necrosis, chromatin condensation, and apoptosis. This damage led to the degeneration of several oocytes, in particular under the strongest concentration tested of triclosan (500 ng/L), and thus probably reduced the reproductive function and the fertility of the animal. The deleterious effect of triclosan on the membrane permeability was already highlighted previously [47]. The exposure to triclosan was followed by the violation of the permeability of the membranes of cells and organelles, further leading to haemorrhage and disruption of tissue structures. This type of cytological alteration is the cause of observed tissue weakening in gills and digestive glands, as well as the histological anomalies observed in the current study.

## 5. Conclusions

The multitude of responses in the current experiment produced by measuring several biochemical, histological and physiological end-points proves the importance of comprehensive assessments when evaluating the multifaceted effects of endocrine disruptors following their release in the aquatic habitats. Specifically, the release of triclosan, a widespread endocrine disruptor in aquatic habitats, affected (in the current study) the normal ecophysiology of a common benthic dweller, the clam *R. decussatus*, expressed in lower oxygen consumption and osmoregulation. The toxic effects of triclosan were reflected in two major organs, namely the gills and the digestive glands, where the activity of defence and damage biomarkers increased, but that of neurotoxicity biomarkers was inhibited. Moreover, these biochemical changes were paralleled by changes in the histology of the gills and digestive glands, reflected in inflammatory reactions and malformations of tissues. The wider implications of the current experiment are that ecotoxicological studies also need to cover important biochemical, histological and physiological end-points that are very often intimately correlated and not included in standard laboratory bioassays.

## Figures and Tables

**Table 1:** Intensity of histopathological alterations of gills of *Ruditapes decussatus* exposed to triclosan (C1, C2, and C3) for 3 and 7 days. Incidence of lesions: (−) absent, (+/−) sometimes, (+) frequent, (++) very frequent, (+++) always present. Ut: untreated; C1: 100 ng/L; C2: 200 ng/L; C3: 500 ng/L.

Histopathological Alterations	After 3 Days				After 7 Days			
	Ut	C1	C2	C3	Ut	C1	C2	C3
Increase of the hemocytic number (AH)	+/−	+/−	+	++	+/−	+/−	+++	+++
Hemocytic infiltration (IH)	+/−	+/−	++	++	+/−	+/−	+++	+++
Alteration of the lamellar epithelium (AE)	+/−	+/−	++	++	+/−	+/−	+++	+++
Erosion of eyelashes (EC)	−	+/−	++	++	−	+/−	++	+++
Lamellar alteration (AL)	−	+/−	+	++	−	+/−	++	++
Lamellar fusion (FL)	−	−	+/−	+	−	+/−	++	++
Rupture of filaments (DF)	−	+/−	+	+	−	+/−	+	++
Irregularity of the interfilamentous space (IEI)	−	+/−	+	++	+/−	+/−	+	++
Vacuolization (V)	−	−	+	+	−	−	+/−	+
Index of Damage (ID)	0.15	0.4	1.25	1.6	0.2	0.45	1.95	2.3

**Table 2:** Intensity of histopathological alterations at digestive glands of *Ruditapes decussatus* exposed to triclosan (C1, C2, and C3) for 3 and 7 days. Incidence of lesions: (−) absent, (+/−) sometimes, (+) frequent, (++) very frequent, (+++) always present. Ut: untreated; C1: 100 ng/L; C2: 200 ng/L; C3: 500 ng/L.

Histopathological Alterations	After 3 Days				After 7 Days			
	T	C1	C2	C3	T	C1	C2	C3
Hemocytic infiltration (IH)	+	+	++	+++	+	+	+++	+++
Increasing of hemocytic number (AH)	+	+	++	+++	+	+	+++	+++
Light dilation (LD)	−	+/−	+	++	−	+/−	++	+++
Shrinkage of epithelial cells (RE)	−	+/−	+	++	−	+/−	+	++
Swelling of epithelial cells (GE)	−	−	+	+	−	−	+	++
Cellular debris (DC)	−	−	+	++	−	−	+	++
Cellular necrosis (NC)	−	−	+/−	+	−	+/−	+	+
Tubular necrosis (NT)	−	−	+/−	+	−	+/−	+	+
Alteration of the conjunctive tissue (AT)	+/−	+/−	+	+	+/−	+/−	++	++
Elongation of the digestive tubes	−	−	−	+/−	−	−	+/−	+
Damaged nucleus	−	+/−	+	+	−	+/−	+	+
Vacuolization	+/−	+	+	++	+/−	+	++	++
Abundant secretion	+/−	+	+	++	+/−	+	+++	+++
Index of Damage (ID)	0.26	0.46	1	1.65	0.26	0.53	1.65	2

## Data Availability

All the data in the article are available from the corresponding author upon reasonable request.
